# Conservative management by embolization of a ruptured renal arterio-venous malformation (AVM) in Hereditary Hemorrhagic Telangiectasia (HHT)

**DOI:** 10.1186/s42155-024-00444-8

**Published:** 2024-03-16

**Authors:** Romain L’Huillier, Gaële Pagnoux, Sophie Dupuis-Girod, Nicolas Stacoffe

**Affiliations:** 1 Department of Diagnostic and Interventional Radiology, Hospices Civils de Lyon, University of Lyon, Pavillon B, Hôpital Edouard Herriot, 5 Place D’Arsonval, Lyon, 69003 France; 2 LabTAU - Inserm U1032, Lyon, 69003 France; 3 The French Comprehensive Liver Center, Hospices Civils de Lyon, University of Lyon, Lyon, 69004 France; 4 Genetics Department and National, Hospices Civils de Lyon, HHT Reference Center Hôpital Femme-Mère-Enfant, Bron, 69677 France; 5 Laboratory Biology of Cancer and Infection, Inserm, CEA, Université Grenoble Alpes, Grenoble, France; 6 Department of Diagnostic and Interventional Radiology, Centre Hospitalier Lyon Sud, Hospices Civils de Lyon, University of Lyon, Pierre-Bénite, 69495 France

**Keywords:** Embolization, Rendu-Osler-Weber disease, Arterio-Venous Malformation, Hematuria, Hereditary Hemorrhagic Telangiectasia

## Abstract

**Background:**

Renal arteriovenous malformation (AVM) in Hereditary Hemorrhagic Telangiectasia (HHT) is uncommon and only few cases have been described, mainly with surgical management because of uncontrolled hematuria.

**Case presentation:**

We managed a 70-year-old patient with HHT who presented with hematuria and left flank pain. Computed Tomography and ultrasound showed left renal AVM of 18 mm with clotting in the urinary tract. An external ureteral catheter was placed during 3 days to allow rinsing and facilitate elimination of clots.

Given the patient's hemodynamic stability, a non-surgical management was chosen. Treatment of the AVM was performed by trans-arterial embolization using micro-coils and ethylene–vinyl alcohol copolymer.

**Conclusions:**

Our case study shows a conservative management by embolization of ruptured left renal AVM revealed by hematuria in a 70-year-old patient with HHT.

## Background

Hereditary hemorrhagic telangiectasia (HHT) is an autosomal dominant disease characterized by recurrent spontaneous epistaxis, cutaneous and mucosal telangiectases, and visceral arteriovenous malformations (AVMs) that may affect many organs, especially the lungs, liver, digestive tract and brain. Clinical diagnosis is based on the Curaçao criteria (recurrent epistaxis, telangiectases, visceral lesions and family history) and is considered definite if three criteria are fulfilled [1]. In 96% of patients with a definite clinical diagnosis of HHT, a mutation is identified in one of these 3 genes: endoglin (ENG, HHT type 1), activin receptor-like kinase-1 (ACVRL1, HHT type 2), and Mothers against decapentaplegic homolog 4 (SMAD4, juvenile polyposis–HHT overlap syndrome) [2].

Visceral AVMs most often involve the lungs, liver and brain [3]. Renal involvement in HHT is uncommon [4] and only few cases have been described [5–7] and have required surgical management for uncontrolled hematuria [5, 7].

This case describes a non-operative management by embolization of a left renal AVM revealed by hematuria in a 70 years old patient with HHT.

## Case presentation

A 70-year-old patient with HHT type 2 (activin receptor-like kinase-1, ACVRL1 gene mutation identified) followed at the French National HHT Reference Center (Hôpital Femme-Mère-Enfant, Hospices Civils de Lyon, France) was transferred to the Edouard Herriot University Hospital (Hospices Civils de Lyon, France) from a peripheral center for spontaneous hematuria with abdominal left side pain.

Clinical diagnosis of HHT was done in 2005 by using Curacao criteria [1]: recurrent spontaneous epistaxis, cutaneous telangiectases, familial history and severe hepatic involvement complicated by high cardiac index (4.7L/min/m2).

At the admission, hemoglobin was 13,6g/dl, platelets count was 240,000 /µl, creatinine level was 63µmol/l with glomerular filtration rate of 85mL/min.

**Fig. 1:**
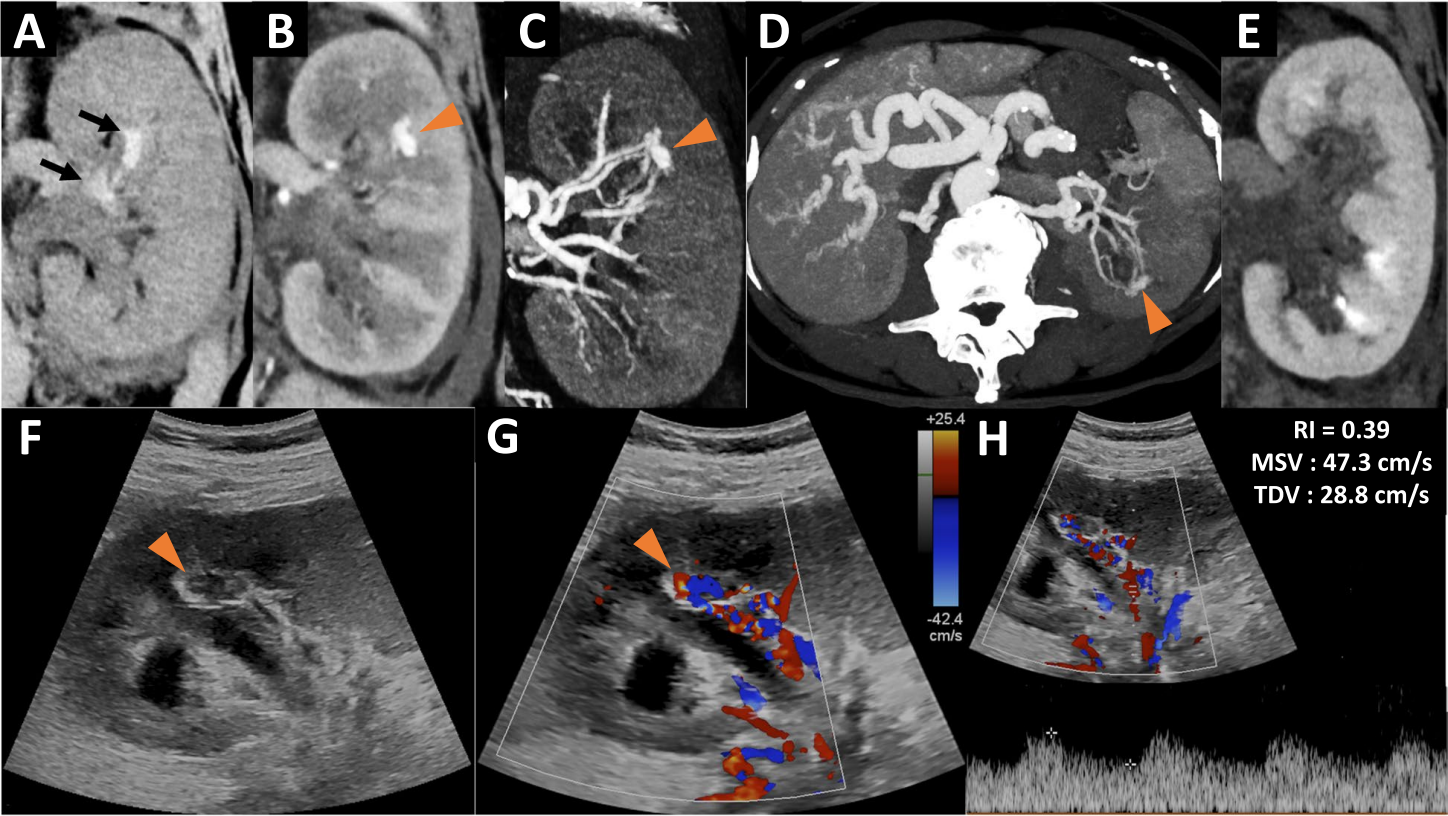
Computed tomography, ultrasound and doppler of left renal AVM (**A**) Unenhanced CT in coronal reconstruction showing spontaneous hyperdensities in the superior calyx and renal pelvis of the left kidney, corresponding to clotting (black arrows), with dilatation of the left upper urinary tract **B**, **C** & **D** Contrast-enhanced CT at the cortical phase in coronal (**B**), coronal MIP 15mm and axial MIP 20mm showing the left renal AVM (orange arrowhead) with several arterial feeders. Axial MIP reconstruction shows the liver involvement of HHT with hepatic artery > 6mm and distal subscapular telangiectases **E** Contrast–enhanced CT at the excretory phase showing a delay in urinary excretion due to blood clots, obstructing the left upper urinary tract **F** & **G**—B mode ultrasound (**F**) and doppler (**G**) showing left renal AVM as an hypoechoic, well-marginated lesion, close to the sinus (orange arrowhead). Arterial feeders are seen on doppler examination **H** – Pulsed doppler in an arterial feeder of the AVM showing reduced resistance index (IR = 0,39) with increased tele-diastolic velocity (TDV≈29cm/sec)

Abdominopelvic computed tomography (CT) was performed in order to explore left side pain associated with hematuria and showed a dilatation of the left upper urinary tract (grade 2 hydronephrosis) with perirenal fluid effusion caused by left ureteral clotting (Fig. 1A). Contrast-enhanced CT showed a left renal AVM appearing as well-marginated renal vascular lesion of 18mm that enhances similar to the blood pool with early enhancement at the cortical phase (Fig. 1B, C, and D). On the CT scan, at least two arterial feeders were found (Fig. 1C, and D). There was no active bleeding in the perirenal space or in upper urinary tract. At the excretory phase (Fig. 1E), there was a delayed excretion from the left kidney, associated with clotting in the upper urinary excretory tract (Fig. 1A). Also, known hepatic vascular abnormalities were unchanged. On chest CT (not shown), there was no pulmonary AVM.

On ultrasound, left renal AVM appeared as a well-marginated hypoechoic region in the renal parenchyma (Fig. 1F). Doppler examination showed a high flow vascular lesion (Fig. 1G) with reduced resistance index in arterial afferences (Fig. 1H).

As the patient was clinically stable, a non-operative management was preferred. The patient underwent external ureteral catheter placement for water rinsing during 3 days to promote clots elimination. After removal of the external ureteral catheter we decided to perform a trans-arterial embolization of the left renal AVM.

For catheterization, we used a 4-French Cobra 2 (C2) catheter and a 2.4-French straight microcatheter. The procedure was performed under sedation and local anesthesia.

**Fig. 2:**
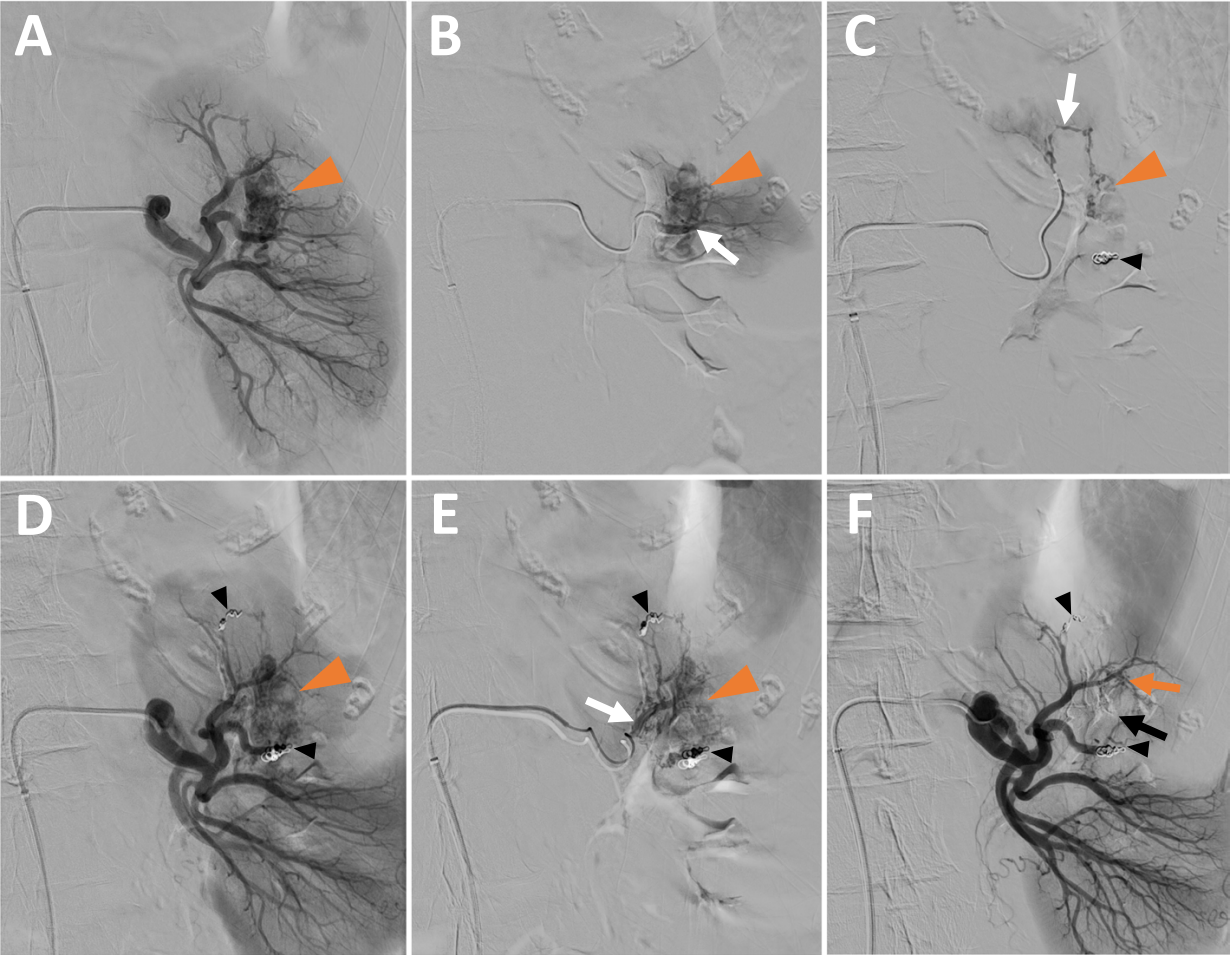
Trans-arterial embolization of left renal AVM **A** – Left renal arteriography showing the AVM (orange arrowhead) with nidus. Arterial feeders are difficult to individualized **B**, **C** & **D** – Microcatheterization of interlobar and arcuate arterial branches (white arrows) and embolization of 2 arterial branches feeding the AVM (orange arrowhead) with 2 detachable micro-coils (blacks arrowheads).Control left renal arteriography (**D**) showing persistent opacification of the AVM nidus (orange arrowhead). **E** – Microcatheterization of an interlobar artery (white arrow) feeding the AVM (orange arrowhead) and embolization by 0,8ml of Ethylene–Vinyl alcohol (EVOH) copolymer. **F** Control left renal arteriography showing a close-to-complete occlusion of the AVM nidus using 2 micro-coils (black arrowheads) and EVOH copolymer (black arrow). At the end of the procedure, a very small portion of the circulating nidus remains (orange arrow), which we have chosen not to treat in order to avoid more extensive renal devascularization

Left renal angiography revealed no active arterial bleeding or pseudoaneurysm but a small complex high-flow AVM with a nidus and 3 main arterial feeders (Fig. 2A, B, C, D, and E). This AVM was treated by embolization using 2 detachable (3 mm and 2mm diameter) micro-coils (Concerto Helix; Medtronic, Minneapolis, MN, USA) (Fig. 2C, and D) and 0,8ml of ethylene–vinyl alcohol (EVOH) copolymer (Onyx® LES, Covidien, Plymouth, MN) (Fig. 2E, and F).

Post embolization arteriography confirmed the close-to-complete occlusion of the AVM with partial (25%) left renal devascularization (Fig. 2F).

48 h after embolization patient’s creatinine level was 53µmol/l, hemoglobin was 13,2g/dl. There was no recurrence of macroscopic hematuria, the patient remained hemodynamically stable was discharged 4 days after embolization.

## Discussion

Lungs and liver are the organs most frequently affected by vascular abnormalities in HHT; while renal involvement is uncommon [4].

Apart from HHT, renal AVMs are rare and often congenital or post-traumatic or even iatrogenic after renal biopsy. Macroscopic hematuria is the most frequent clinical manifestation [8] and occurs when a dysplastic vessel breaks into the urinary collecting system.

Imaging (ultrasound, CT and MRI) usually enables non-invasive diagnosis of renal AVMs, distinguishing them from simple arteriovenous fistulas by the presence of a nidus with, in most cases, several tortuous feeder arteries [8].

The management of AVMs revealed by hematuria depends on the intensity of the hematuria and its consequences. Severe hematuria can be life-threatening at short term, and may require surgical management by nephrectomy. Low-flow hematuria can lead to clotting and obstruction of the upper urinary tract, causing flank pain and a risk of secondary infection, which may require urine drainage.

In hemodynamically stable patients, trans-arterial embolization has been described as effective and various embolizing agents have been used (absorbable gelatin sponge, absolute alcohol, polyvinyl alcohol, coils, n-butyl 2-cyanoacrylate (nBCA) glue and ethylene–vinyl alcohol (EVOH) copolymer) [9, 10]. In our case, we chose a combined strategy with the use of micro-coils to occlude two arterial feeders and reduce inflow in the AVM; and then 0,8ml of ethylene–vinyl alcohol (EVOH) copolymer in the last arterial feeder in order to reach the nidus and occlude almost completely the AVM.

We chose EVOH copolymer rather than nBCA because it precipitates much more slowly avoiding the danger of incomplete embolization and the risk of rapid reflux and non-target embolization. In patients with HHT and renal AVMs, extreme care must be taken when using liquid embolization agents: the risk of the liquid agent migrating into the renal vein, and therefore the risk of pulmonary embolization, must be taken into account when there are associated pulmonary AVMs, since there is then a risk of systemic arterial embolization. It is therefore probably preferable for embolization of renal AVMs in patients with HHT to be performed in an expert center.

## Conclusion

Our report describes a renal arteriovenous malformation (AVM) in a patient with Hereditary Hemorrhagic Telangiectasia (HHT) type 2, revealed by macroscopic hematuria and successfully treated by embolization. This clinical report is original because complicated renal AVMs are extremely rare in HHT and successful non-surgical management has been very rarely described previously.

## Abbreviations

AVM: Arterio-Venous Malformation
HHT: Hereditary Hemorrhagic Telangiectasia
CT: Computed Tomography
MRI: Magnetic Resonance Imaging
EVOH: Ethylene–Vinyl alcohol
nBCA: N-butyl 2-cyanoacrylate
